# The Doctors' Wives Association

**Published:** 1914-07-04

**Authors:** 


### The Doctors' Wives Association.

We have now the pleasure to announce that arrangements have been made by which members who have joined the Association will be able, through its introduction, to obtain many articles. They include :—

Every-day Requisites, under twelve sub-headings.

Outdoor and Indoor Sports and Games, covering many necessary and essential requisites for sport, and all the games now so popular in this country.

Household requirements, including many of the chief essentials, under fourteen sections.

Another great division consists of Men's Clothing.

Arrangements have been made with a Dress Expert, who will give advice without charge to members requiring any article of ladies' dress, such as gowns, blouses, millinery, underclothing, and lingerie of all kinds.

Finally, many Garden and Stable requisites may be obtained by members of the Association, together with Farm and Agricultural Machinery and Implements, Saddlery, Harness, and all Horse Gear.

Full particulars and a detailed list and information will be sent to each member of the Association on or before July 14.

We may add that members who are resident in the country and decide to come to London to make their own selection, by advising the Association in advance and stating what their requirements are, will be supplied with introductions to enable them to do their own shopping on the best terms.

Members' privileges will commence on July 15, 1914.

